# Hemolytic mechanism and virulence of Antarctic psychrophilic *Pseudomonas*

**DOI:** 10.3389/fmicb.2026.1959947

**Published:** 2026-10-06

**Authors:** Chaoyue Yang, Zhenyu Liu, Xiaoya Peng, Li Zhou, Zhixiong Xie, Jun Huang, Wenhui Jiang, Songtao Ai, Fang Peng

**Affiliations:** 1 China Center for Type Culture Collection (CCTCC), College of Life Sciences, Wuhan University, Wuhan, China; 2 Animal Biosafety Level III Laboratory at Center for Animal Experiment, Wuhan University School of Medicine, Wuhan, China; 3 Key Laboratory of Polar Environment Monitoring and Public Governance, Ministry of Education, Wuhan University, Wuhan, China; 4 Hubei Key Laboratory of Cell Homeostasis, College of Life Sciences, Wuhan University, Wuhan, China; 5 Chinese Antarctic Center of Surveying and Mapping, Wuhan University, Wuhan, China

**Keywords:** climate change, hemolysis, polar regions, *Pseudomonas*, virulence

## Abstract

Polar warming is expected to release and redistribute psychrophilic microorganisms, thereby increasing their likelihood of encountering warmer environments and potential hosts. However, it remains uncertain whether psychrophilic polar bacteria maintain pathogenic properties relevant to mammalian hosts during such transitions. Here, we investigated two hemolytic Antarctic *Pseudomonas* isolates, 3S6 and NS21, assigned to *P. antarctica* and *P. yamanorum*, respectively. Both isolates exhibited hemolytic activity, biofilm formation, and siderophore production, although these phenotypes were weaker than those of *P. aeruginosa*. Functional analyses indicated that their cell-associated hemolytic activity was dependent on the ExlBA two-partner secretion system (TPS), in which the outer-membrane transporter ExlB secretes the pore-forming cytotoxin ExlA: deletion of *exlB* abolished hemolysis, whereas genetic complementation restored this phenotype, and this activity was temperature-sensitive, consistent with repression of ExlBA expression under elevated temperatures. Temperature elevation from 20 °C to 28 °C reduced hemolytic activity, *exlBA* transcription, and intracellular cAMP levels, while strongly inducing *cpdA*, implicating a CpdA-cAMP/Vfr-associated regulatory mechanism. Elevated temperature also triggered a transition from a motile to a biofilm-associated lifestyle in strain 3S6, potentially reflecting adaptive resource allocation toward survival rather than virulence under thermal stress. Under host-relevant conditions, both isolates caused ExlBA-dependent membrane damage in mammalian cells and shortened *Caenorhabditis elegans* survival. In mice, they caused transient bloodstream invasion and acute lung inflammation without mortality or sustained colonization. Collectively, these findings indicate that psychrophilic hemolytic polar *Pseudomonas* possess restricted, temperature-responsive host-damaging capacity under exposure conditions associated with environmental warming.

## Graphical abstract

**Figure:**
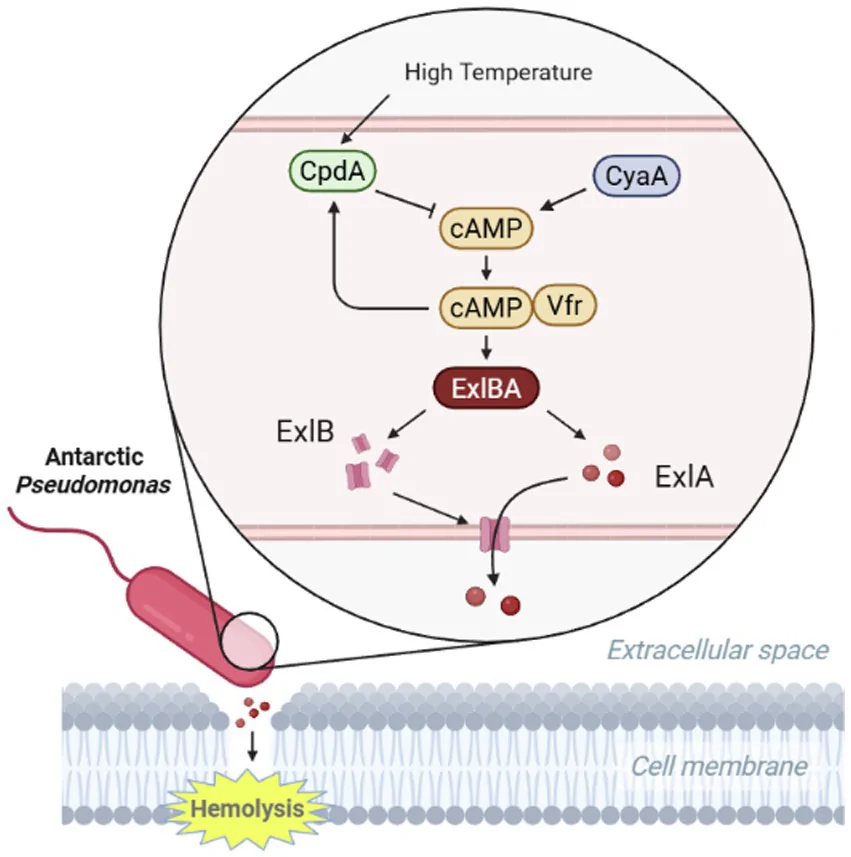

## 1 Introduction

Global warming is reshaping polar ecosystems by altering cryosphere stability, meltwater connectivity, and biological interactions (Antoni et al., 2020; Wang et al., 2025). The Arctic is warming faster than the global average, whereas Antarctic warming is regionally heterogeneous, with pronounced changes reported in the Antarctic Peninsula (Prates and Vieira, 2023; Rantanen et al., 2022; Wang et al., 2019). Glacier retreat, permafrost thawing, and episodic warming events may release psychrophilic microorganisms from previously isolated cryospheric habitats and increase their dispersal across newly connected environments (Ashton et al., 2017; Dawson et al., 2023). These changes do not necessarily imply the emergence of established pathogens, but they raise a testable question: whether warming-associated exposure and temperature shifts may reshape the physiological activity and virulence-related traits of psychrophilic environmental opportunists.

Polar microorganisms have been increasingly recognized as reservoirs of traits linked to environmental persistence and opportunistic host interaction. Genomic surveys have identified virulence factor-encoding genes in Antarctic and Arctic isolates, including genes encoding adhesion factors, motility-related proteins, secretion systems, and putative hemolytic determinants (Arros et al., 2024; Fang et al., 2024). Hemolytic bacteria capable of growth at 37 °C, including *Bacillus*, *Micrococcus*, and *Pseudomonas* species, have also been recovered from Arctic environments (Mogrovejo et al., 2020). Among these taxa, *Pseudomonas* is particularly relevant because members of this genus possess traits related to motility, biofilm formation, extracellular enzyme production, biosurfactant synthesis, secretion systems, and stress tolerance (de Sousa, 2025; Jurado-Martin et al., 2021; Silby et al., 2011). However, the virulence potential of psychrophilic, hemolytic *Pseudomonas* strains from polar regions has remained largely unexplored.

Temperature-dependent virulence in *Pseudomonas* is increasingly understood as phenotype-specific rather than uniformly enhanced by warming. For example, a Ysc-family T3SS in psychrotrophic *P. lundensis* is active at 37 °C and further induced in the murine intestine (Ravi et al., 2022), whereas in the tundra isolate *P. fragi* D12, fimbrial gene expression, motility, and biofilm formation show distinct temperature optima (Li et al., 2026). These findings indicate that different virulence-linked traits may follow distinct thermal response curves. Compared with highly host-specialized secretion systems, hemolysis provides a particularly useful entry point because it is directly measurable, reflects membrane-damaging potential, and can represent a broad opportunistic trait relevant to both environmental competition and host-cell injury. Hemolytic activity has also been reported in Arctic bacterial isolates, including *Pseudomonas* examined across environmental and host-relevant temperatures (Mogrovejo et al., 2020). Yet the molecular basis of hemolysis in psychrophilic polar *Pseudomonas*, and more critically, whether and how their virulence is regulated by temperature and is deployable against animal hosts, remains largely unexplored.

*Pseudomonas* can produce hemolysis or membrane damage through multiple mechanisms, including biosurfactants, phospholipases, and protein toxins (Barker et al., 2004; Deng et al., 2017; Engel and Balachandran, 2009; Reis et al., 2011). Among these mechanisms, the ExlBA two-partner secretion system (TPS) is particularly relevant because ExlA is a pore-forming cytotoxin described in T3SS-negative *Pseudomonas* strains, providing a cell-associated route of membrane damage distinct from diffusible hemolytic factors (Elsen et al., 2014; Huber, 2022). In *P. aeruginosa* models, *exlBA* transcription is activated by the CyaB-cAMP/Vfr pathway, but this activation is not temperature-sensitive (Berry et al., 2018; Huber, 2022). However, whether ExlBA-like determinants occur in psychrophilic polar *Pseudomonas*, whether they contribute functionally to hemolysis, and whether their expression is temperature responsive remain unresolved.

Here, an investigation was conducted on two representative psychrophilic hemolytic polar isolates, *P. antarctica* 3S6 and *P. yamanorum* NS21, to examine their hemolytic characteristics, underlying molecular mechanisms, and responses to temperature shifts, as well as to evaluate their virulence potential. Genomic, molecular, and functional approaches were employed to explore the possible involvement of the ExlBA two-partner secretion system in hemolytic activity, its temperature-dependent regulation across cold environmental, intermediate permissive, and host-entry thermal conditions, and the broader virulence-associated phenotypes of these isolates. This study aimed to elucidate the molecular basis and temperature-dependent regulation of hemolysis in polar *Pseudomonas*, and to assess their virulence potential under host-relevant conditions, thereby informing trait-specific risk assessment of polar microorganisms under climate change.

## 2 Materials and methods

### 2.1 Ethics statement

The animal experiments were performed in the Animal Biosafety Level-2 (ABSL-2) Laboratory and approved by the Institutional Animal Care and Use Committee at College of Life Sciences, Wuhan University (SKLV-AE2025 009, SKLV-AE2025 022). All animal studies were conducted in accordance with the ARRIVE guidelines. The protocols and procedures for handling infectious pathogens in the ABSL-2 Laboratory facility were approved by the Institutional Biosafety Committee (IBC) at the State Key Laboratory of Virology and Biosafety. All the samples were inactivated according to IBC-approved standard procedures for the removal of specimens from high containment.

### 2.2 Strains and cultivation conditions

The strains and the plasmids used in this study are described in Supplementary Table S1. *Pseudomonas* strains 3S6 and NS21 were isolated from soil samples collected from the Fildes Peninsula, Antarctica. The strains were grown in liquid Luria-Bertani (LB) medium (hopebio, HB0128) at 20 °C. *Escherichia coli* strains were grown in liquid LB medium at 37 °C, of which DH5α and S17-1(λpir) required supplementation with 50 μg/mL kanamycin (aladdin, K331597).

Assay temperatures were selected to span the thermal contexts relevant to the environmental release, dispersal, and host contact of polar microorganisms: 10 °C and 20 °C represent permissive temperatures for Antarctic psychrophilic *Pseudomonas* (20 °C also being the routine cultivation temperature used throughout this study), 28 °C approximates an upper thermal boundary that cold-adapted isolates may transiently encounter during warming-associated dispersal toward temperate terrestrial or coastal habitats, and 37 °C represents the body temperature of mammalian hosts. This gradient allowed each virulence-associated phenotype to be assessed across cold environmental, intermediate permissive, and host-relevant thermal conditions.

### 2.3 Genetic manipulations

The plasmids utilized in this study and the primers used for PCR are listed in Supplementary Tables S2, S3. Gene knockout *Pseudomonas* strains were constructed using the homologous recombination principle (Hmelo et al., 2015). The upstream fragment and downstream fragment of the target gene were amplified via PCR using specific primers. The two PCR products were amplified by fusion PCR, digested, and ligated with T4 DNA Ligase (TaKaRa, 2011A) into the digested plasmid pK18mobsacB (Schafer et al., 1994). The recombinant plasmid was transformed into *E. coli* S17-1(λpir) and subsequently transferred into wild-type strains by conjugation. Next, the pK18mobsacB was removed using a 15% (w/v) sucrose no-salt LB (NSLB) plate. Mutants were further confirmed by PCR using primers of the target gene and were stored at −80 °C for later use.

Complementation of the target gene in *Pseudomonas* strains was achieved using the broad-host-range plasmid pBBR1MCS-2 (Kovach et al., 1995). The target gene was amplified by PCR and cloned into the pBBR1MCS-2 vector. The recombinant plasmid was also transformed into *E. coli* S17(λpir) and then transferred into the recipient strain through conjugation. Transconjugants were selected on VBMM agar plates (Hmelo et al., 2015) containing 50 μg/mL kanamycin. Positive clones verified by colony PCR were cultured in liquid medium supplemented with 50 μg/mL kanamycin and stored at −80 °C for later use.

### 2.4 Comprehensive virulence profiling

*Motility assay*: Bacterial cultures were standardized to an OD_600_ = 0.2. Two microliter of samples were inoculated in the center of the swarming medium (LB, 0.5% glucose, 0.5% agar) (Jia et al., 2024) and swimming medium (LB, 0.3% agar) (Rickard et al., 2024). The plates were incubated at 20 °C for 24 h, and the migration zones were determined. The center of the twitching medium (LB, 1% agar) was punctured with a sterilized toothpick, then 2 μL of samples were added to the wells. After incubation at 20 °C for 5 d, agar was carefully removed, and the plastic petri dish was stained with 0.1% crystal violet (Sinopharm, 71012314) for 15 min (Kar et al., 2024). The migration zones were determined.

*Proteolytic activity*: The *Pseudomonas* strains were inoculated in the center on of the skim milk-NSLB agar plates with 10% w/v skimmed milk (BD, 232100) and incubated at 20 °C for 48 h (Lobo et al., 2024).

*Pyocyanin and fluorescein production*: The *Pseudomonas* strains were cultured on *Pseudomonas* F agar (Hopebio) medium at 20 °C for fluorescent pigments testing. Luminescence was determined for each culture under ultraviolet light (360 ± 20 nm) (Duman et al., 2020). Pyocyanin production was tested on *Pseudomonas* P agar (Hopebio).

*Biofilm assay*: Biofilm formation was quantified by crystal violet staining (Vargas-Cuebas et al., 2024). Overnight LB-grown bacterial cultures were diluted in fresh medium (1:100) and incubated in 96-well culture plates at 20 °C for 24 h allowing stable biofilm formation. The firmly attached biofilm was then washed twice with phosphate buffered saline (PBS) to eliminate planktonic cells. Further, 200 μL methanol (Sinopharm, 10014108) was poured to individual well and left for 15 min. Then, 200 μL of 0.1% (v/v) crystal violet was added to the wells of the air-dried plate and kept for 15 min. The crystal violet stain was discarded while the stained biofilms were washed twice thoroughly with PBS and air-dried. The stained biofilm was then dissolved into 200 μL of 95% ethanol (Sinopharm, 10009218) and the absorbance was determined at 570 nm in a microplate reader (BioTek). Finally, the measured values were normalized against the bacterial cell density (OD_600_).

*Siderophores production*: Added the filtered supernatant to an equal volume of CAS test solution (Coolaber, PM0821-2), and incubated for 30 min in the dark. The absorbance was determined at 630 nm in a microplate reader (BioTek) (Zeng et al., 2022).

### 2.5 Whole genome sequencing

*Pseudomonas* cultures were collected by centrifugation and washed with PBS three times. The whole genome was sequenced via a second-generation Illumina platform and third-generation Nanopore sequencing technology. Genome assembly was performed using Unicycler (v 0.5.0) (Wick et al., 2017) with a hybrid approach: First, high-accuracy Illumina reads (Q30 > 85%) were assembled to generate a high-quality bacterial genomic contigs. Subsequently, Nanopore long reads were used to bridge these contigs into complete genomes. Finally, Pilon was employed to further polish the assembly using the short-read data, yielding a genome with enhanced accuracy.

The assembled genome was annotated using the NCBI Prokaryotic Genome Annotation Pipeline (PGAP). This pipeline predicted protein-coding genes (CDSs) using GeneMarkS-2, assigned functional annotations based on homology searches against the RefSeq and Conserved Domain Database (CDD), and identified tRNA, rRNA, and other non-coding RNA genes. The final annotation results were output in GenBank format. In addition to this standard annotation, potential virulence factors were specifically annotated by performing blastp analysis of all CDSs against the Virulence Factors of Pathogenic Bacteria database (VFDB, http://www.mgc.ac.cn/VFs/) (Chen et al., 2005).

### 2.6 Cell-associated hemolysis assay

Sheep erythrocytes (Sbjbio, SBJ-RBC-S003) and *Pseudomonas* strains were washed three times in PBS and resuspended in RPMI-1640 medium (MeilunBio, MA0553) without pH indicator. And then, the assays were started by mixing 100 μL of sheep erythrocytes and 100 μL of bacteria (multiplicity of infection, MOI = 1), which were centrifuged at 100 × *g* for 10 min at 4 °C and incubated at 37 °C for 2 h to assess hemolytic activity under host-relevant thermal conditions (Sperandio et al., 2010). For osmoprotection assays, sheep erythrocytes were incubated with 30 mM osmoprotectants. After that, the mixtures were centrifuged at 10,000 × *g* for 5 min at 4 °C to separate the hemoglobin released from erythrocytes. The negative controls were noninfected sheep erythrocytes, and the positive control was obtained by inducing complete lysis by adding 0.1% SDS (BioFroxx, 3250GR500). The absorbance was measured at 560 nm. %Hemolysis = [X-B/T-B] × 100, while X represent the treated or untreated samples, B represent the negative control and T represent the positive control.

### 2.7 Quantitative real-time PCR analysis

Total RNA of *Pseudomonas* was extracted by *SteadyPure* Universal RNA Extraction Kit (Accurate Biology, AG21017) according to the manufacturer’s instructions. The RNA was converted to cDNA by reverse transcriptase using *Evo M*-*MLV* RT Mix Kit (Accurate Biology, AG11728). The synthesized cDNA was used as a template for quantitative real-time PCR (qRT-PCR) analysis by SYBR Green Premix *Pro Taq* Kit (Accurate Biology, AG11701). To assess the relative expression of *exlA*, *exlB*, *cpdA*, *cyaA*, and *vfr* mRNAs, their expression levels were normalized against an endogenous reference (*rpoD*) by the comparative threshold cycle method.

### 2.8 cAMP measurements

*Pseudomonas* cultures were grown to an OD_600_ of 0.2. Subsequently, 5 mL of bacterial cells were harvested, and intracellular cAMP levels were quantified using a cAMP ELISA Kit (Sangon, D770001) according to the manufacturer’s instructions. The absorbance was measured at 450 nm and normalized against the bacterial cell density.

### 2.9 Transcriptomic analysis

Strain 3S6 grown on LB-blood agar at different temperatures (20 °C and 28 °C) for about 3 d, were harvested and sent to Majorbio Biopharm Technology Co., Ltd. for transcriptomic sequencing. Because lawn cultures cannot be standardized by optical density, comparability between the 20 °C and 28 °C conditions relies on this identical cultivation and harvesting protocol, on normalization of the sequencing data during bioinformatic analysis, and on the consistency of the transcriptional responses with the phenotypic assays performed with standardized liquid-culture inocula. The DEGs were screened using the DESeq2 (v 1.42.0) software based on an absolute fold change>2 with a *p*-value<0.05. After that, DEGs were mapped to terms in gene ontology (GO, http://www.geneontology.org/) (Ashburner et al., 2000) and Kyoto Encyclopedia of Genes and Genomes (KEGG, http://www.genome.jp/kegg/) (Ogata et al., 1999) database for functional and pathway analysis. The VFDB were used for virulence genes annotation.

### 2.10 Microscopic analysis

Strains were cultivated in 12-well microplates containing circular glass coverslips at 20 °C or 28 °C for 24 h. After incubation, the culture supernatant was carefully removed, and the adherent biofilms on the coverslips were gently washed with PBS. The biofilms were then stained with 0.1% acridine orange (AO) (Beyotime, C0233S) for 15 min in the dark. After staining, excess dye was removed by washing with PBS. The stained biofilms were visualized using a confocal laser scanning microscope (CLSM) (Zeiss). Imaging was performed using a 488 nm argon laser for excitation.

### 2.11 Cell culture and cytotoxicity assay

The epithelial lung carcinoma cell line A549 (CCTCC GDC0063) and the murine macrophage cell line J774A.1 (CCTCC GDC0176) were cultured in Dulbecco’s modified Eagle medium (DMEM) (Gibco, C11995500BT), supplemented with 10% fetal bovine serum (FBS) (Zhejiang Tianhang Bio, Every Green, HB0128) and 1% penicillin/streptomycin (P/S) (Yeasen, 60162ES76), at 37 °C, in 5% CO_2_. The human colorectal adenocarcinoma cell line DLD-1 (CCTCC GDC0620) was cultured in Roswell Park Memorial Institute (RPMI) 1,640 medium (Gibco, C11875500BT) supplemented with 10% FBS and 1% penicillin/streptomycin, at 37 °C, in 5% CO_2_.

The day before the infection, cells were plated in 96-well plates (10,000 cells/well) and incubated for 1 night. Two hour before infection, medium was replaced by basal medium without FBS or P/S. The infection was done at MOI = 20, 8 h. The level of LDH released in the cell culture supernatant was detected by LDH cytotoxicity assay detection kit (Beyotime, C0017) following the manufacturer’s instructions.

### 2.12 *Caenorhabditis elegans* survival assay

*Caenorhabditis elegans* survival assays were performed using the wild-type N2 Bristol strain. Synchronized eggs were collected within 2 h and cultured on NGM plates at 20 °C until the L4 stage. *Pseudomonas* strains and *E. coli* OP50 were adjusted to an OD_600_ of 0.2, concentrated 10-fold, and seeded onto NGM plates. OP50 served as the negative control. L4 nematodes were randomly assigned to each bacterial treatment, with 100 worms per group. To prevent progeny development, NGM plates containing 0.1 mM FUDR were used during the assay. Worms were transferred to fresh plates every 96 h and monitored daily for survival. Death was defined as the absence of movement and pharyngeal pumping after gentle touch.

### 2.13 Mouse infection assay

*Pseudomonas* and *E. coli* K-12 cells were harvested, washed, and resuspended in PBS. Six to eight weeks old female BALB/c mice (Biont) were anesthetized and then intranasally inoculated with 1 × 10^7^ CFU of bacteria per mouse once on d0. Mice in the control group received an equal volume of sterile PBS.

At 1 and 2 days post-infection, mice were euthanized by carbon dioxide asphyxiation. Lung tissues and blood samples were collected aseptically. The lungs were homogenized in PBS. Bacterial loads in homogenates and blood were determined by plating serial 10-fold dilutions and counting CFU. On day 2 post-infection, lung tissues from each group were fixed in 4% paraformaldehyde (Sinopharm, 80096618), embedded in paraffin, sectioned, and subjected to hematoxylin and eosin (H&E) staining for pathological examination. Serum and lung homogenates also collected on day 2 were further analyzed for TNF-α, IL-1β, and IL-6 levels by ELISA (youkelife, EM0010/EM0029/EM0004).

### 2.14 Statistical analysis

All experiments were performed in at least triplicate. The data shown in the figures are means ± standard deviation (SD) of the replicates. Statistical differences were determined using one-way analysis of variance (ANOVA) or *t*-test in GraphPad Prism 8, with *p*-value<0.05 considered statistically significant.

## 3 Results

### 3.1 Genomic characteristics of hemolytic *Pseudomonas* strains 3S6 and NS21

Two hemolytic psychrophilic bacterial isolates, 3S6 and NS21, were isolated from Antarctic soil samples and initially screened for erythrocyte-lysing activity on blood agar plates. Both isolates exhibited pronounced β-hemolysis, indicating the potential for membrane-damaging activity (Figure 1A). Direct contact between bacterial cells and erythrocytes produced strong cell-associated hemolysis, with efficiencies of 78.86 and 90.96% for strains 3S6 and NS21, respectively (Figure 1B). Whole-genome sequencing revealed that strains 3S6 and NS21 possessed circular chromosomes of 6.27 Mb and 7.18 Mb, respectively, while highly conserved GC contents (Table 1). Gene annotation identified 5,594 and 6,565 coding sequences (CDSs), indicating higher genomic complexity in NS21. Phylogenomic analyses, including 16S rRNA gene identity, average nucleotide identity (ANI) and digital DNA–DNA hybridization (dDDH), assigned 3S6 to *Pseudomonas antarctica* and NS21 to *Pseudomonas yamanorum*, demonstrating their affiliation with established Antarctic *Pseudomonas* lineages (Figure 1C and Table 2). Comparative genomic profiling revealed that both isolates harbor a repertoire of virulence-associated genes related to adhesion, motility, biofilm formation, secretion systems, and exoenzymes. 3S6 and NS21 possess a suite of virulence factors at levels intermediate between clinical *P. aeruginosa* strains and other pathogenic *Pseudomonas* species (Figure 1D). While their overall gene content is not as extensive as that of highly pathogenic *P. aeruginosa* (PAO1, PA14, ST235), both polar isolates exhibit relatively high representation of effector delivery system and motility-related genes, suggesting the retention of functional modules that may support host interaction or environmental competitiveness. Other categories, including exoenzymes, toxins, and immune modulators, are present at moderate to low levels, consistent with a conditional opportunistic potential.

**Figure 1:**
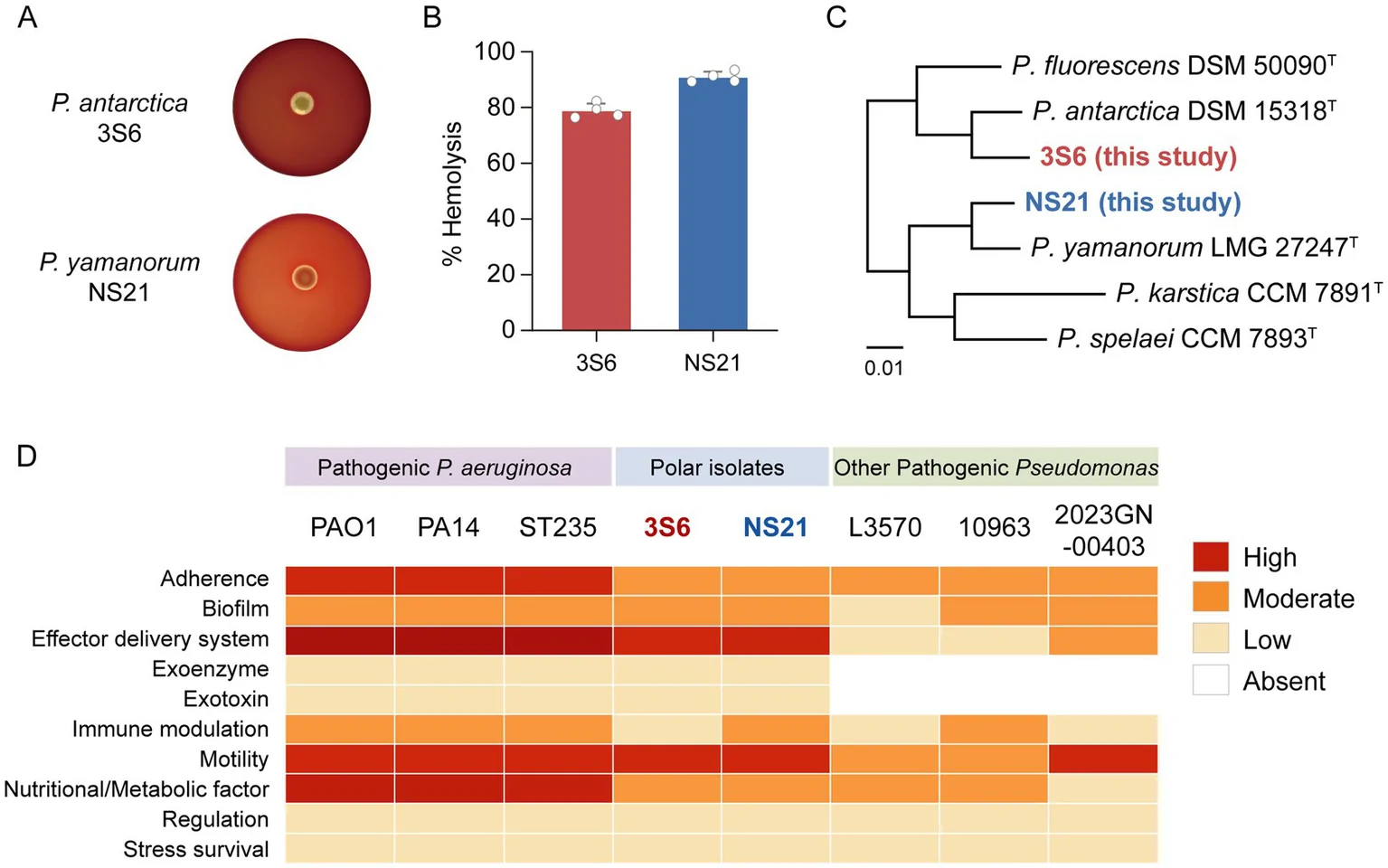
Hemolytic characterization, taxonomic identification, and virulence-associated genomic features of Antarctic *Pseudomonas* isolates 3S6 and NS21. **(A)** Representative β-hemolytic phenotypes of *Pseudomonas antarctica* 3S6 and *Pseudomonas yamanorum* NS21 on blood agar containing 10% sheep blood. **(B)** Quantitative analysis of cell-associated hemolysis caused by direct contact between bacterial cells and erythrocytes, MOI = 1. Data are shown as mean ± SD. **(C)** Phylogenomic placement of strains 3S6 and NS21 within representative *Pseudomonas* lineages. Using UBCG 3.0, phylogenetic analysis was performed based on the alignment of 92 core genes. Strain 3S6 clustered with *P. antarctica*, whereas strain NS21 clustered with *P. yamanorum*, supporting their taxonomic assignments based on genome-wide relatedness. **(D)** Comparative heatmap of virulence-associated gene categories among representative pathogenic *P. aeruginosa* strains, polar isolates, and other pathogenic *Pseudomonas* species. The color intensity indicates the relative abundance (high, moderate, low, and absent levels) of genes assigned to each virulence factor category.

**Table 1:** General genomic features of the *Pseudomonas* strains 3S6 and NS21.

Strains	3S6	NS21
Isolation sources	Soils	Soils
Regions	Fildes Peninsula	Fildes Peninsula
Size (bp)	6,272,401	7,182,738
GC content (mol %)	59.27	60.07
Number of gene	5,681	6,652
Number of CDS	5,594	6,565
Number of tRNA	67	67
5S rRNA number	6	6
16S rRNA number	5	5
23S rRNA number	5	5
Number of tmRNA	1	1

**Table 2:** Taxonomic assignment of strains 3S6 and NS21 based on 16S rRNA gene identity, ANI, and dDDH.

Strains	Reference strains	16S rRNA gene identity (%)	ANI (%)	dDDH (%)
3S6	*Pseudomonas antarctica* DSM 15318^T^	99.6	94.8	80.4
NS21	*Pseudomonas yamanorum* LMG 27247^T^	99.7	94.3	75.1

### 3.2 Virulence-associated phenotypes of strains 3S6 and NS21

After confirming the hemolytic activity of these two strains, the next question addressed was to examine a broader set of virulence-associated phenotypes in 3S6 and NS21, guided by genomic analysis. To further evaluate whether 3S6 and NS21 possessed broader virulence-associated phenotypes, proteolytic activity, motility, pigment production, biofilm formation, and siderophore production were compared using *P. aeruginosa* CCTCC AB 2013115, an opportunistic pathogen used here as a phenotypic comparator of each assay.

Plate-based assays showed that both 3S6 and NS21 exhibited proteolytic activity and retained swimming and swarming motility. However, neither strain showed detectable twitching motility. With respect to pigment production, neither 3S6 nor NS21 produced pyocyanin, whereas both strains produced fluorescent pigment (Figure 2A). Quantitative assays further showed that biofilm formation by 3S6 and NS21 was significantly lower than that of *P. aeruginosa* CCTCC AB 2013115 (Figure 2B). A similar reduction was observed for siderophore production: *P. aeruginosa* showed 82.33% PSU, whereas 3S6 and NS21 showed 39.73 and 40.43% PSU, corresponding to approximately 51.74 and 50.89% relative reductions, respectively (*p* < 0.001) (Figure 2C). Together, although the expression levels of various virulence-associated traits in strains 3S6 and NS21 were lower than those of the reference strain CCTCC AB 2013115 under the same experimental conditions, these results nevertheless indicate that 3S6 and NS21 retain multiple virulence-associated traits. *P. aeruginosa* CCTCC AB 2013115 was used as a reference strain and should not be interpreted as a universal standard for the virulence of opportunistic *Pseudomonas*. Therefore, the reduced expression levels of virulence traits in the polar isolates do not imply a lack of pathogenicity; rather, they should be regarded as the retention of a partial, environmentally adapted repertoire of virulence-associated functions.

**Figure 2:**
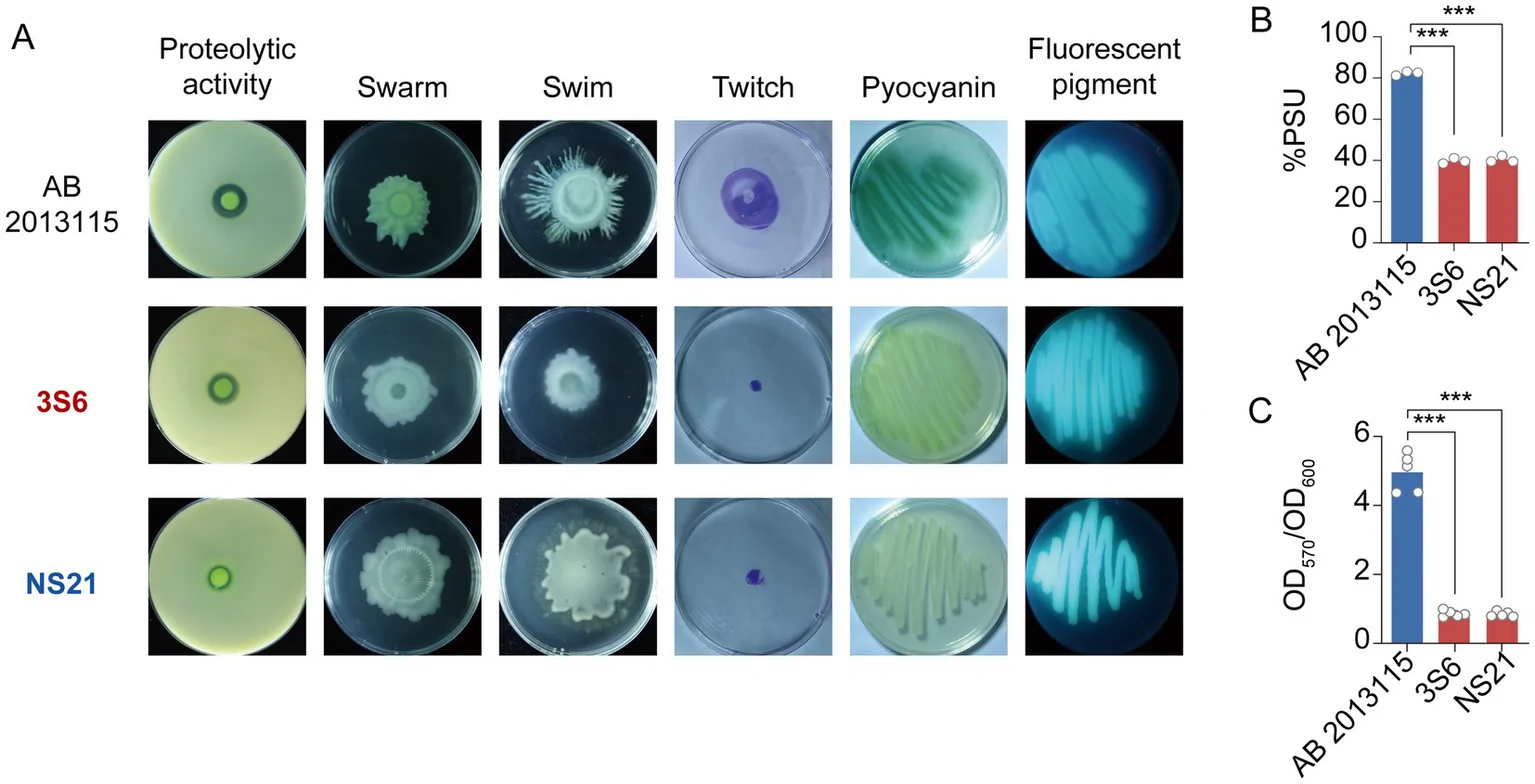
Virulence-associated phenotypes of 3S6 and NS21 compared with *P. aeruginosa*. **(A)** Representative plate-based assays showing proteolytic activity, swarming motility, swimming motility, twitching motility, pyocyanin production, and fluorescent pigment production in 3S6, NS21, and *P. aeruginosa* CCTCC AB 2013115. **(B)** Quantitative analysis of siderophore production, expressed as percent siderophore units (% PSU). **(C)** Quantitative analysis of biofilm formation, expressed as OD_570_/OD_600_. Data are shown as mean ± SD. ns, not significant; *p < 0.05, **p < 0.01, ***p < 0.001.

### 3.3 ExlBA system as the hemolytic determinant in *Pseudomonas* strains 3S6 and NS21

The contact-dependent phenotype (Figure 1B) suggested that the responsible toxin was surface-associated rather than secreted freely into the medium, implying that the hemolytic virulence factors may be associated with TPS systems or T3SS (Basso et al., 2017; Dacheux et al., 2001; Elsen et al., 2014; Sperandio et al., 2010). The addition of PEG 800 reduced the hemolytic activity of strain 3S6 by 63.98%, while PEG 1000 reduced the hemolytic activity of strain NS21 by 57.41% (Figure 3A), indicating that the pore-forming virulence protein formed pores of approximately 1.6 nm in diameter, consistent with the exolysin ExlA previously described in *Pseudomonas* (Figure 3B) (Basso et al., 2017).

**Figure 3:**
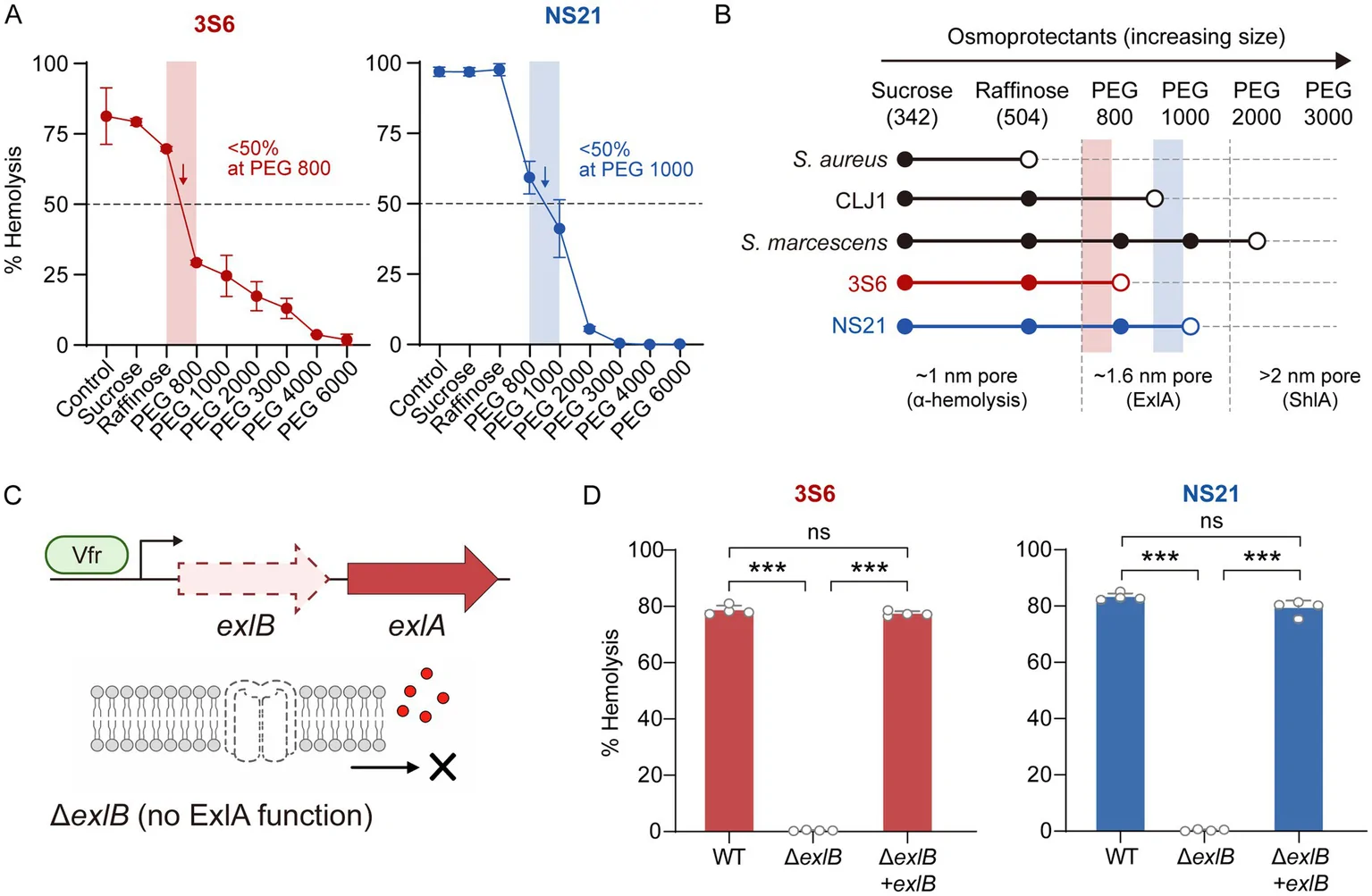
The ExlBA two-partner secretion system mediates cell-associated hemolysis in *Pseudomonas* strains 3S6 and NS21. **(A)** Osmotic protection assays showing the effects of osmoprotectants with increasing molecular sizes on erythrocyte lysis by strains 3S6 and NS21. The hemolysis assays performed with 3S6 or NS21 in the presence of 30 mM sugars or PEG osmoprotectants. The dashed line indicates 50% hemolysis. **(B)** Schematic summary of osmotic protection profiles and inferred pore-size ranges. The inhibitory profiles of 3S6 and NS21 were consistent with pore formation in the approximate size range reported for ExlA-mediated hemolysis (Basso et al., 2017), rather than smaller *α*-hemolysin-like pores (Menestrina et al., 2003) or larger ShlA-like pores (Hertle et al., 1999). **(C)** Schematic representation of the *exlBA* locus and the functional consequence of *exlB* deletion. ExlB functions as the outer-membrane transporter required for ExlA secretion or surface-associated activity; deletion of *exlB* is expected to prevent ExlA-dependent membrane damage. **(D)** Hemolytic activity of wild-type, *exlB*-deletion, and complemented strains. Bacteria were cultured at 20 °C and incubated with sheep erythrocytes at MOI = 1. Data are shown as mean ± SD. ns, not significant; *p < 0.05, **p < 0.01, ***p < 0.001.

The ExlBA two-partner secretion system (TPS), comprising the transporter ExlB and the secreted pore-forming toxin ExlA, required both components for full virulence (Figure 3C) (Bertrand et al., 2020; Huber, 2022; Job et al., 2022; Reboud et al., 2017). To assess the impact of *exlBA* on hemolytic activity, hemolysis assays were performed using wild-type strains (3S6, NS21) and the *exlB*-deletion mutant strains (3S6Δ*exlB,* NS21Δ*exlB*). The results showed that strains lacking ExlB completely abolished hemolytic activity (Figure 3D). Complementation with the *exlB* gene restored the membrane-disrupting activity of the mutant strains, confirming that ExlBA was required for full cytolytic function. These findings demonstrated that the ExlBA system mediated the cell-associated hemolysis in *Pseudomonas* strains 3S6 and NS21.

### 3.4 Temperature elevation attenuates ExlBA-associated hemolysis in association with CpdA induction and reduced cAMP levels

Hemolytic activity in 3S6 and NS21 exhibited a clear temperature-dependent pattern. Both strains formed evident hemolytic zones at 10 °C and 20 °C, whereas hemolysis was markedly attenuated when the incubation temperature was increased to 28 °C (Figure 4A). Quantitative hemolysis assays further confirmed this trend, hemolysis efficiency decreased at least 21.55% (*p* < 0.01) (Figure 4B). Because ExlBA was identified as a major determinant of cell-associated hemolysis in these strains, it was next examined whether the temperature-dependent reduction in hemolysis was associated with altered transcription of the *exlBA* locus. RT-qPCR analysis showed that the transcript levels of both *exlB* and *exlA* were significantly reduced at 28 °C compared with 20 °C in both strains. Specifically, *exlB* expression decreased by 81.24% in 3S6 and 43.76% in NS21, whereas *exlA* expression decreased by 47.47 and 60.96%, respectively (Figure 4C).

**Figure 4:**
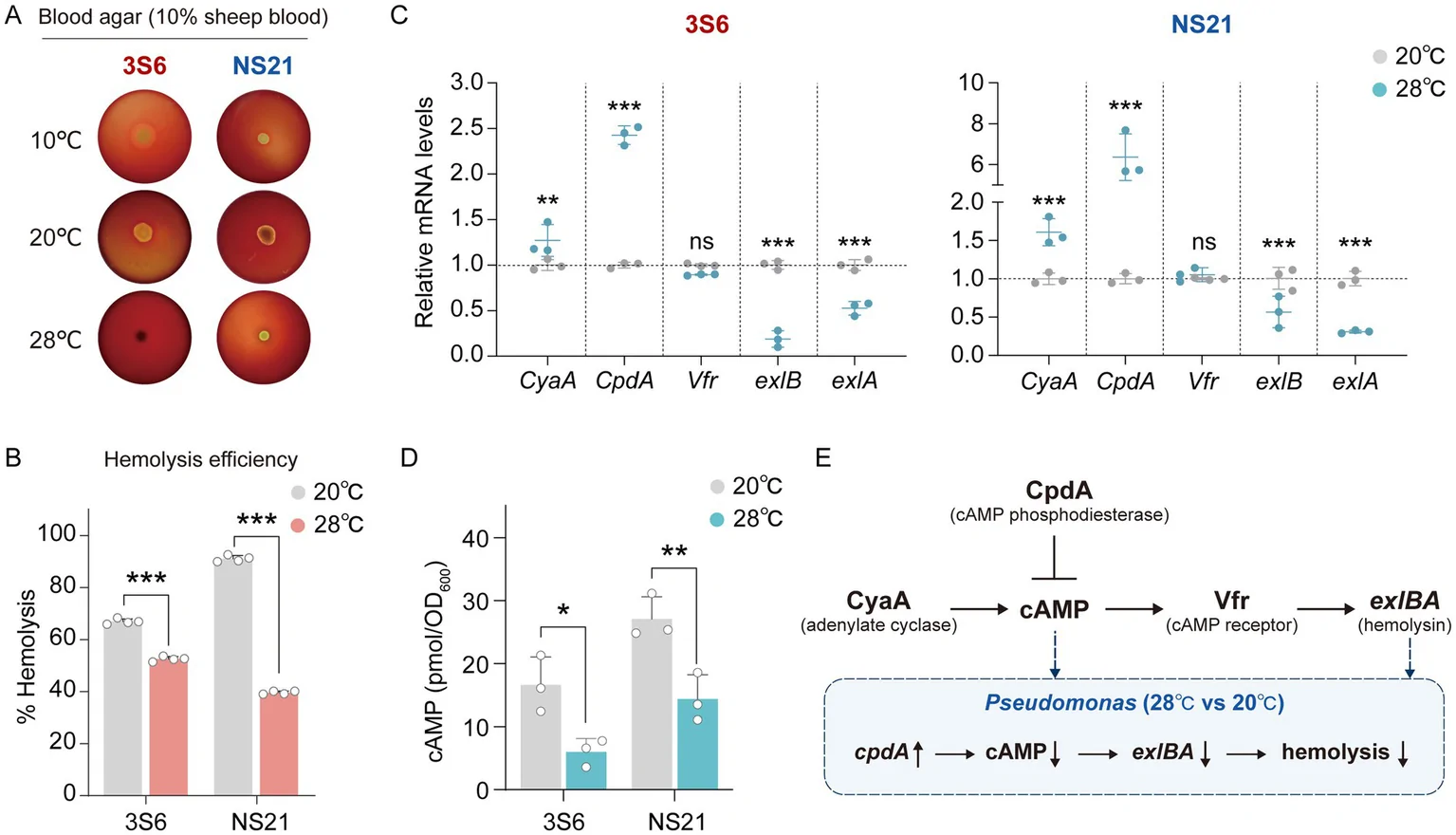
Elevated temperature attenuates hemolysis in association with reduced cAMP levels and decreased *exlBA* transcription. **(A)** Representative hemolytic phenotypes of *Pseudomonas antarctica* 3S6 and *Pseudomonas yamanorum* NS21 on blood agar containing 10% sheep blood after incubation at 10 °C, 20 °C, and 28 °C. **(B)** Quantitative hemolysis efficiency of 3S6 and NS21 at 20 °C and 28 °C. Strains were cultured at 20 °C or 28 °C prior to the assay. The hemolysis assay was performed by incubating *Pseudomonas* cells with sheep erythrocytes at MOI = 1. **(C)** Relative mRNA levels of cAMP/Vfr-related regulatory genes and the *exlBA* locus in 3S6 and NS21 at 20 °C and 28 °C, as determined by RT-qPCR. Expression levels at 20 °C were normalized to 1.0. **(D)** Intracellular cAMP levels in 3S6 and NS21 at 20 °C and 28 °C were measured by enzyme-linked immunosorbent assay (ELISA). **(E)** Proposed model linking temperature-dependent regulation of cAMP metabolism to *exlBA* expression and hemolytic output. At 28 °C, stronger induction of *cpdA* may enhance cAMP degradation, resulting in reduced cAMP availability, decreased *exlBA* transcription, and attenuated hemolysis. Data are shown as mean ± SD. ns, not significant; *p < 0.05, **p < 0.01, ***p < 0.001.

The ExlBA protein is encoded by the *exlBA* operon, and its transcription initiation is regulated by the transcription factor Vfr. Vfr is allosterically activated upon the second messenger cAMP binding, enabling the complex to recognize and bind specific DNA sites within target promoters (Fuchs et al., 2010; Kanack et al., 2006). Intracellular cAMP levels in *Pseudomonas* are primarily balanced by adenylate cyclases CyaA and CyaB, and the phosphodiesterase CpdA, which synthesize and degrade cAMP, respectively (Almblad et al., 2015; Jin et al., 2019) (Figure 4E). However, the *cyaB* homolog has not been annotated in the genomes of these specific *Pseudomonas* strains, it was hypothesized that temperature-dependent hemolysis may be mediated through the CpdA-cAMP-Vfr regulatory axis.

Therefore, to validate this regulatory pathway, the transcript levels of *vfr*, *cyaA*, and *cpdA* under different temperature conditions were quantified by qRT-PCR. Although alterations in temperature did not induce a significant change in the mRNA level of *vfr*, two cAMP metabolism-related genes, *cyaA* and *cpdA*, were significantly upregulated at 28 °C (*p* < 0.01), with *cpdA* showing a much stronger induction than *cyaA*. In 3S6, *cpdA* and *cyaA* were upregulated by 2.43-fold and 1.27-fold (*p* < 0.01), respectively, whereas in NS21, they were upregulated by 6.36-fold and 1.61-fold (*p* < 0.001), respectively (Figure 4C). Consistent with this expression pattern, intracellular cAMP levels were significantly reduced at 28 °C, showing a 1.88- to 3.11-fold decrease (*p* < 0.05) (Figure 4D).

On the basis of these observations, we propose a working model in which elevated temperature upregulates *cpdA* transcription; the resulting enhancement of CpdA-mediated cAMP turnover is expected to lower intracellular cAMP availability and, in turn, the formation of the active cAMP-Vfr complexes required for efficient *exlBA* transcription, ultimately attenuating hemolytic output (Figure 4E). This thermoregulatory cascade provides a molecular basis for temperature-responsive virulence control in polar *Pseudomonas*.

### 3.5 Elevated temperature induces broad physiological reprogramming in psychrophilic *Pseudomonas*

Because strain 3S6 completely lost visible hemolytic activity on blood agar at 28 °C (Figure 4A), whereas strain NS21 retained a reduced but detectable hemolytic zone, transcriptomic analysis was performed using strain 3S6 to further investigate the physiological responses associated with temperature elevation. Bacterial lawns grown on blood agar at 20 °C and 28 °C were collected, and three biological replicates were subjected to RNA sequencing (Figure 5A).

**Figure 5:**
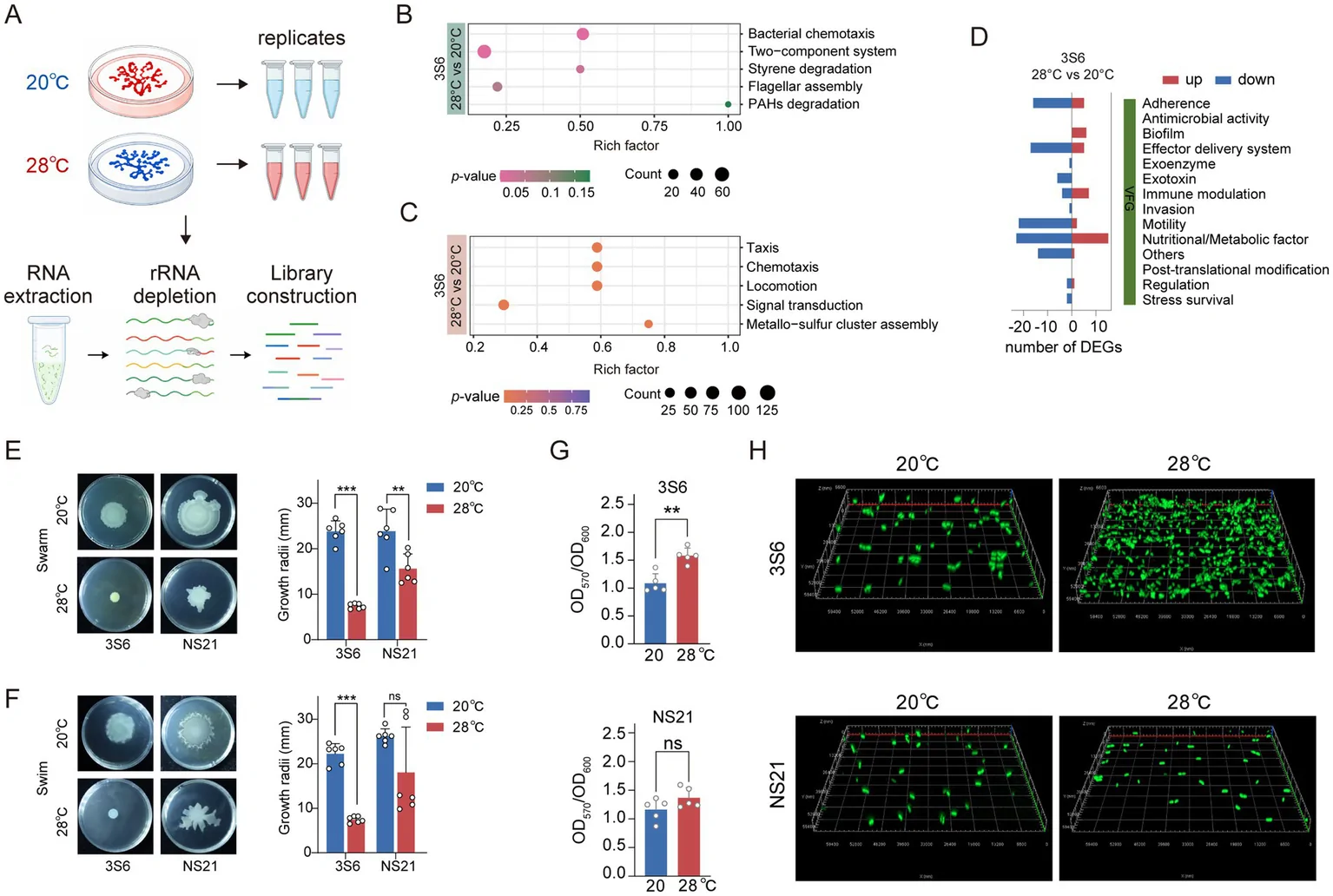
Elevated temperature induces broad physiological reprogramming and a motile-to-biofilm lifestyle shift in strain 3S6. **(A)** Experimental design of the transcriptomic analysis. Strain 3S6 was grown on LB-blood agar at 20 °C or 28 °C (three biological replicates per condition) and subjected to RNA sequencing. **(B)** Gene ontology (GO) enrichment analysis of differentially expressed genes (DEGs; |fold change| > 2, *p* < 0.05) between 28 °C and 20 °C. **(C)** Kyoto Encyclopedia of Genes and Genomes (KEGG) pathway enrichment analysis of the DEGs. **(D)** Expression changes of VFDB-annotated virulence-associated genes at 28 °C vs. 20 °C, grouped by functional category. **(E)** Representative images of swarming and swimming motility of 3S6 and NS21 at 20 °C and 28 °C. **(F)** Quantitative analysis of the swarming and swimming migration zones. **(G)** Quantitative biofilm formation (crystal violet staining; OD_570_/OD_600_) of 3S6 and NS21 at 20 °C and 28 °C. **(H)** Representative confocal laser scanning microscopy images of acridine orange-stained biofilms formed by strain 3S6 at 20 °C and 28 °C. Data are shown as mean ± SD. ns, not significant; *p < 0.05, **p < 0.01, ***p < 0.001.

Functional enrichment analyses of differentially expressed genes consistently indicated that elevated temperature primarily affected motility-related functions, including bacterial chemotaxis, locomotion, flagellar assembly, and signal transduction pathways (Figures 5B,C). To further examine virulence-associated responses, differentially expressed genes were annotated against VFDB. Elevated temperature was associated with widespread downregulation of genes related to adherence, effector delivery systems, motility, and nutritional or metabolic factors, whereas biofilm-associated genes were predominantly upregulated (Figure 5D). These findings suggest that elevated temperature not only attenuates virulence-associated traits but may also reduce the overall metabolic activity of strain 3S6.

Previous studies have shown that type IV pili-mediated motility and adhesion facilitate ExlBA-dependent cytotoxicity in *Pseudomonas* (Basso et al., 2017). However, neither 3S6 nor NS21 exhibited twitching motility (Figure 2A), and genome annotation further revealed the absence of a complete type IV pili system (Table 3). These observations suggest that flagellum-associated motility and biofilm formation may represent key determinants of host contact and surface attachment in these psychrophilic isolates. To test this hypothesis, motility and biofilm formation were compared between 20 °C and 28 °C in both strains. Elevated temperature dramatically reduced both swarming and swimming motility in strain 3S6, whereas NS21 retained partial motility under the same conditions (Figures 5E,F). This may explain why NS21 still exhibited a hemolytic zone on blood agar plates at elevated temperatures. In contrast, crystal violet assays and confocal microscopy demonstrated a marked increase in biofilm formation by strain 3S6 at 28 °C, while no significant changes were observed in NS21 (Figures 5G,H).

**Table 3:** Presence and absence of core type IV pilus genes in hemolytic polar *Pseudomonas* strains.

Functional module	Gene	3S6	NS21
Major pilin	*pilA*	+	+
Extension motor	*pilB*	−	−
Secretin pore	*pilQ*	+	+
Retraction motor	*pilT*	+	+
Retraction accessory motor	*pilU*	−	−
Inner membrane platform	*pilC*	+	+
Alignment complex	*pilM/pilN/pilO/pilP*	−	−
Prepilin peptidase	*pilD*	−	−

“+” indicates the presence of the annotated gene, whereas “−” indicates that the gene was not identified in the genome annotation.

These findings indicate that elevated temperature induces a shift from a motile to a biofilm-associated lifestyle in strain 3S6, potentially limiting host contact efficiency and contributing to reduced ExlBA-mediated hemolytic activity.

### 3.6 Polar isolates retain transient activity at 37 °C and induce ExlBA-dependent host-cell membrane damage

Because these polar isolates are not typical mesophilic pathogens, after the ExlBA-associated hemolytic mechanism and its temperature-dependent regulation were defined, it was next assessed whether they could transiently retain growth-related activity and hemolytic potential after exposure to a host-relevant temperature.

To assess whether 3S6 and NS21 could transiently retain biological activity at a host-relevant temperature, bacterial suspensions grown at 20 °C were adjusted to OD_600_ = 0.2 and shifted to 37 °C. OD_600_ and hemolytic activity were then monitored at 6, 12, and 24 h (Figure 6A). The two strains displayed distinct OD_600_ dynamics at 37 °C. NS21 showed a continuous increase in OD_600_ over 24 h, reaching 0.52, whereas the OD_600_ of 3S6 declined after 6 h, suggesting that 3S6 retained growth-related activity only transiently at 37 °C (Figure 6B). In contrast to the OD_600_ profiles, hemolytic activity decreased rapidly in both strains as the duration of exposure to 37 °C increased. This pattern is consistent with the previous observation that elevated temperature attenuated *exlBA* transcription and reduced hemolytic activity.

**Figure 6:**
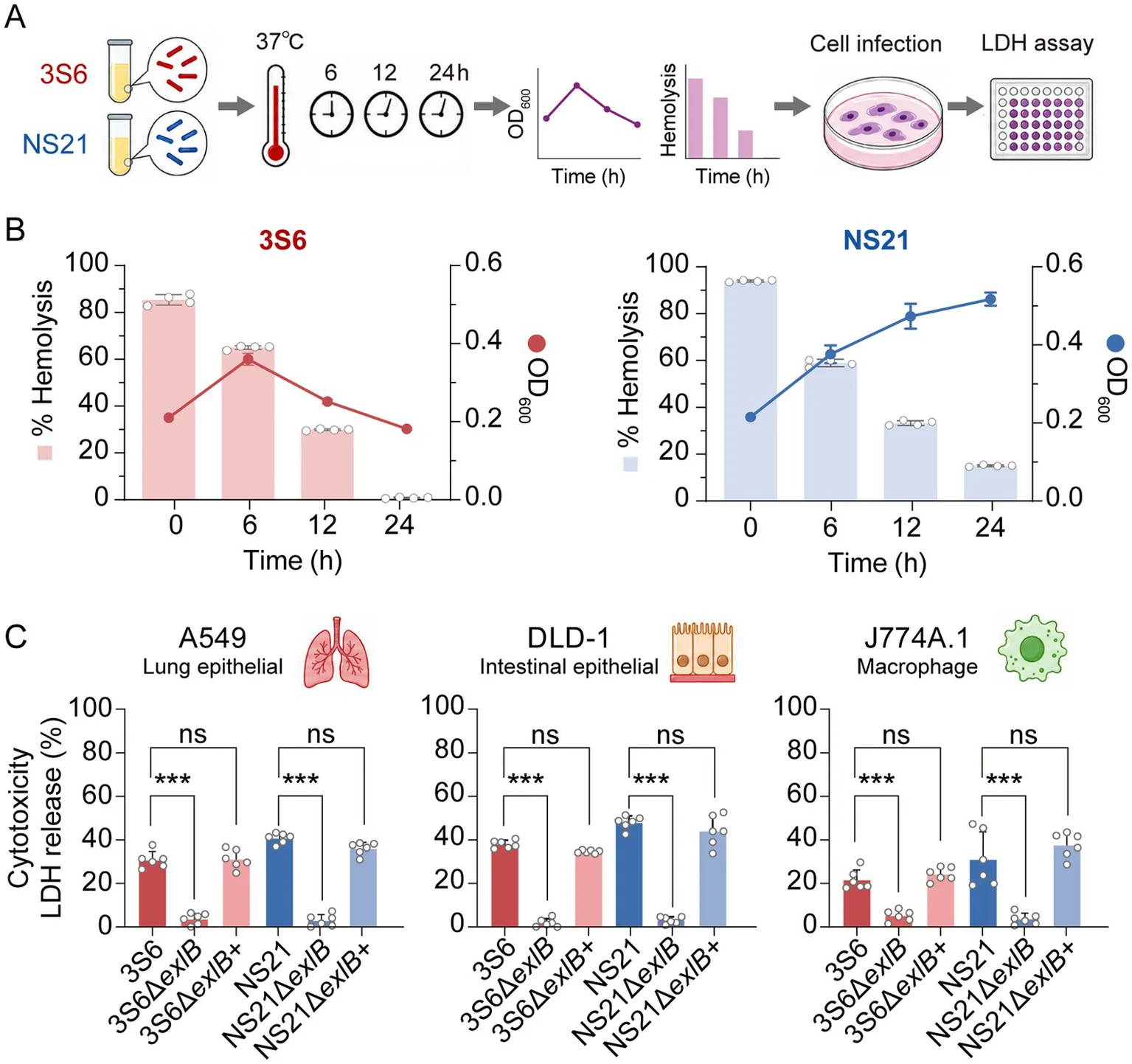
Transient activity at 37°C and ExlBA-dependent cytotoxicity of 3S6 and NS21 toward host cells. **(A)** Schematic overview of the 37°C temperature-shift assay and host-cell infection assay. Bacterial suspensions grown at 20 °C were adjusted to OD_600_ = 0.2 and shifted to 37°C. OD_600_ and hemolytic activity were measured at 6, 12, and 24 h. Host cells were infected for 8 h, and cytotoxicity was assessed by LDH release. **(B)** Changes in hemolytic activity and OD_600_ of 3S6 and NS21 during 37°C exposure. Bars indicate hemolysis efficiency, and lines indicate OD_600_. **(C)** LDH release from A549, DLD-1, and J774A.1 cells infected with wild-type, Δ*exlB*, and *exlB*-complemented strains. Data are shown as mean ± SD. ns, not significant; *p < 0.05, **p < 0.01, ***p < 0.001.

According that both strains retained transient growth-related activity and residual hemolytic potential at 37 °C, their ability to damage host cells was next examined. A549 human alveolar basal epithelial-like cells, DLD-1 human colorectal adenocarcinoma epithelial cells, and J774A.1 mouse macrophage-like cells were infected for 8 h, and cytotoxicity was assessed using an LDH release assay. Infection with either 3S6 or NS21 significantly induced LDH release from all three cell types, with cytotoxicity ranging from 21.61 to 47.96% (Figure 6C). Overall, NS21 induced higher LDH release than 3S6, indicating stronger host-cell membrane-damaging activity.

To further determine the contribution of the ExlBA system to host-cell damage, the cytotoxicity of the wild-type strains, Δ*exlB* mutants, and *exlB*-complemented strains was compared. The Δ*exlB* mutants induced markedly lower LDH release, whereas complementation with *exlB* restored cytotoxicity (*p* < 0.001) (Figure 6C). Together, these results indicated that although 3S6 and NS21 are not typical host-adapted pathogens, they can transiently retain activity at host temperature and mediate ExlBA-dependent membrane damage in host cells.

### 3.7 3S6 And NS21 shorten *Caenorhabditis elegans* survival, with partial ExlBA dependence in NS21

To further assess the *in vivo* virulence of 3S6 and NS21, the *C. elegans* N2 Bristol model was used, and worm survival was compared at 20 °C with *E. coli* OP50 as a negative control and *P. aeruginosa* CCTCC AB 2013115 as a positive control (Figure 7A). Kaplan–Meier survival analysis showed that the positive-control strain CCTCC AB 2013115 was the most virulent, causing obvious worm death as early as 2 d post-exposure and complete mortality by day 14 (Figures 7B,C). In comparison, although the polar isolates 3S6 and NS21 were less virulent than CCTCC AB 2013115, both significantly shortened worm survival and exhibited significantly higher lethality than the negative control OP50 (mean lifespan, 19.26 ± 4.84 d; *p* < 0.001).

**Figure 7:**
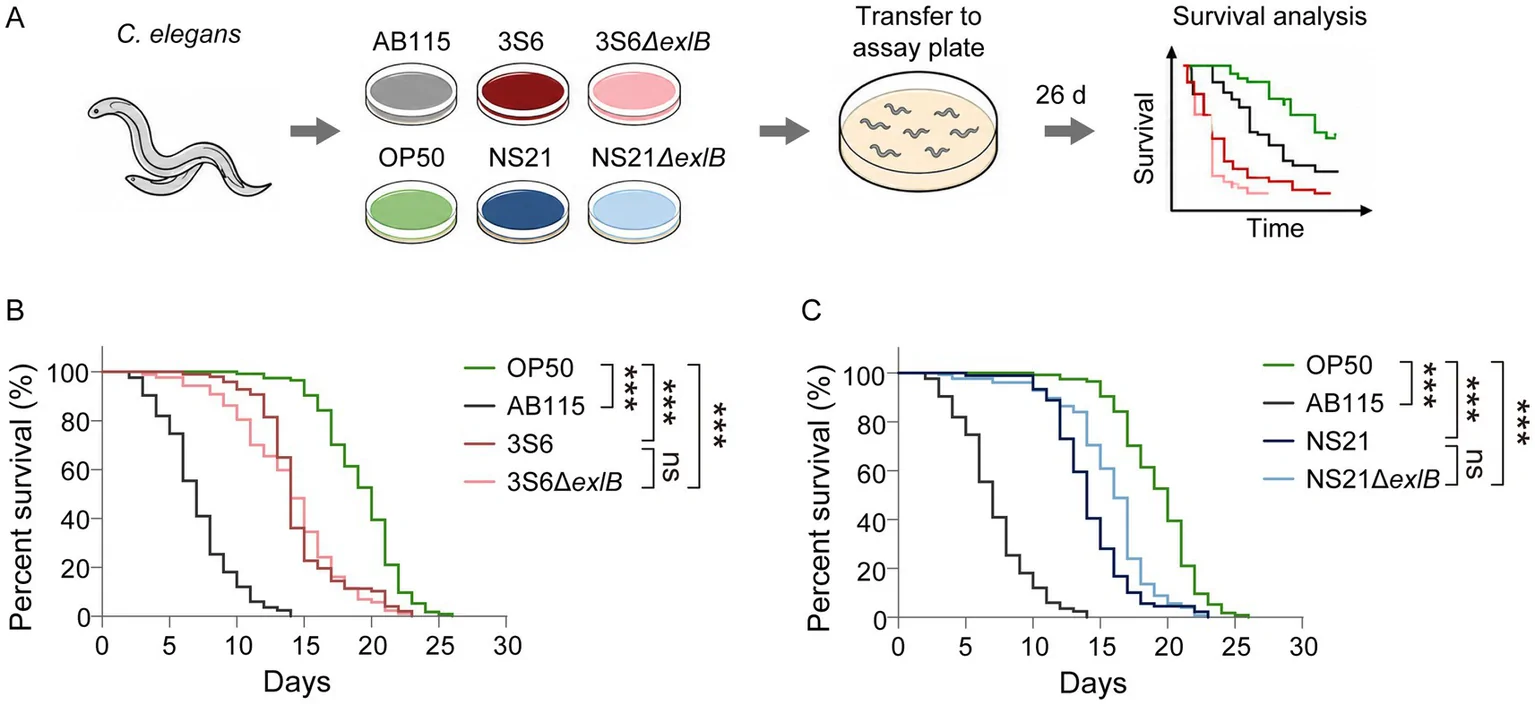
Nematocidal activity of 3S6 and NS21 in the *C. elegans* model. **(A)** Schematic overview of the *C. elegans* survival assay. Synchronized L4-stage N2 Bristol worms were exposed to bacterial lawns and maintained at 20 °C for survival analysis over 26 d. *E. coli* OP50 and *P. aeruginosa* CCTCC AB 2013115 were used as negative and positive controls, respectively. **(B)** Survival curves of worms exposed to OP50, CCTCC AB 2013115, 3S6, or 3S6Δ*exlB*. **(C)** Survival curves of worms exposed to OP50, CCTCC AB 2013115, NS21, or NS21Δ*exlB*. Survival was analyzed by the Kaplan–Meier method. Statistical significance was determined by the log-rank test. ns, not significant; *p < 0.05, **p < 0.01, ***p < 0.001.

The contribution of *exlB* to nematode killing was next examined. Deletion of *exlB* in NS21 significantly attenuated virulence, increasing the mean lifespan of infected worms to 15.43 ± 3.99 d (*p* < 0.001) and prolonging the LT50 from 13.44 d to 15.67 d (Figure 7B). These results indicated that the ExlBA system contributes to NS21-mediated killing in the nematode model. In contrast, no significant difference was observed between 3S6Δ*exlB* and wild-type 3S6 (Figure 7C), suggesting that the contribution of ExlBA to 3S6 virulence is limited or context-dependent *in vivo*. Together with the previous observation that 3S6 showed lower hemolytic activity and weaker cytotoxicity than NS21, this finding further supports strain-specific differences in virulence output.

Notably, all polar Δ*exlB* mutants still caused significantly greater lethality than OP50 (*p* < 0.001) (Figures 7B,C), indicating that ExlBA is not the sole determinant of nematode killing by these polar *Pseudomonas* isolates. Under conditions close to their permissive growth temperature, other secreted factors or metabolic products may also contribute to *in vivo* toxicity.

### 3.8 Polar *Pseudomonas* isolates induce acute lung inflammation despite limited *in vivo* colonization

To further evaluate the infection-related phenotypes of polar *Pseudomonas* isolates in a mammalian host, an acute infection model was established using female BALB/c mice. Mice were intranasally inoculated with 1 × 10^7^ CFU per mouse. Non-pathogenic *E. coli* K-12 and *P. aeruginosa* CCTCC AB 2013115 were used as negative and positive controls, respectively. During the 2-d observation period, mice infected with *P. aeruginosa* CCTCC AB 2013115 died within 1 d, indicating strong *in vivo* virulence (Figure 8A). In contrast, none of the polar isolates caused mortality, but infected mice displayed evident clinical signs, including ruffled and dull fur and reduced activity.

**Figure 8:**
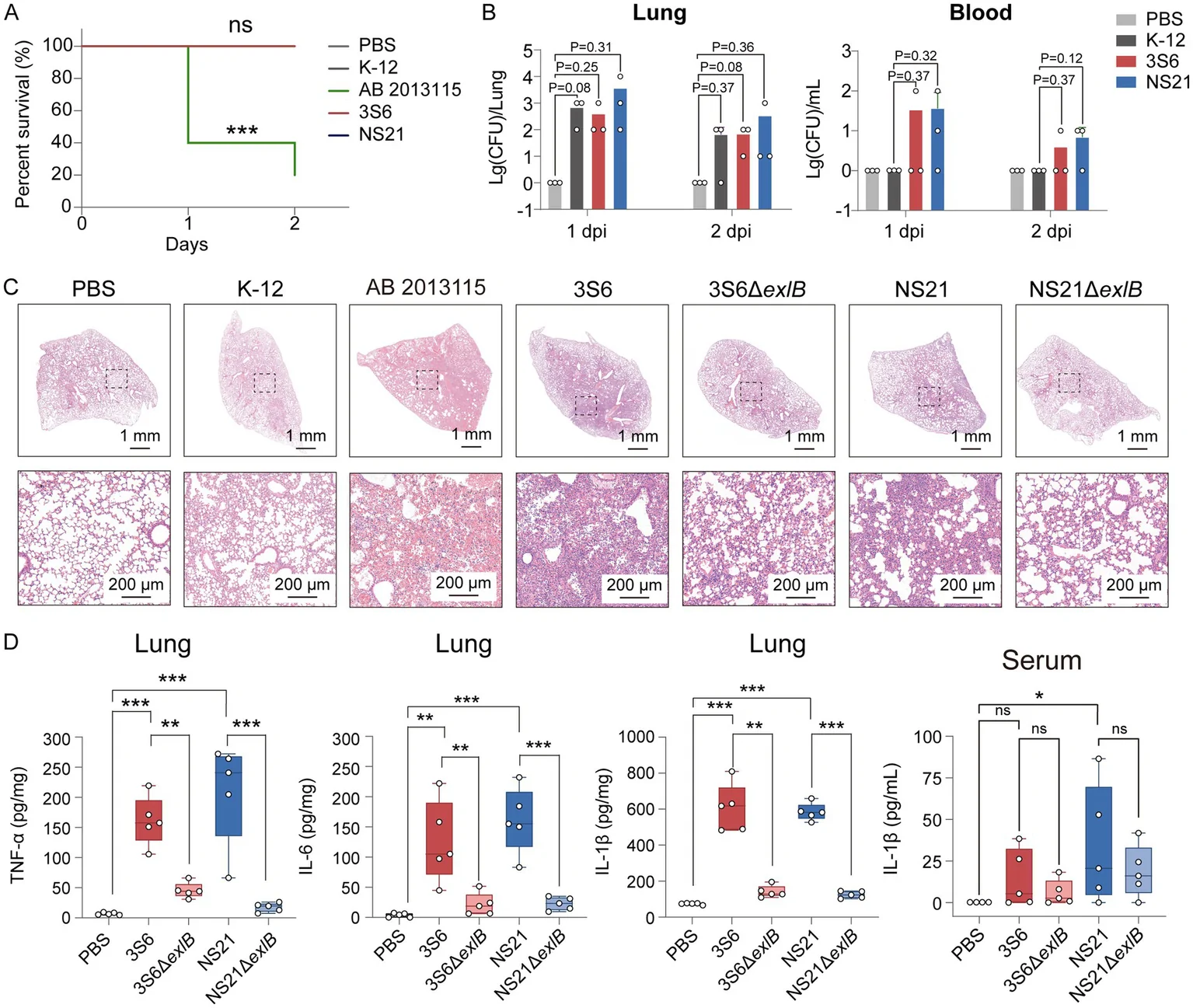
Acute lung inflammation induced by polar *Pseudomonas* isolates in a mouse infection model. Female BALB/c mice were inoculated with 1 × 10^7^ CFU per mouse and monitored for 2 d. *Escherichia coli* K-12 and *P. aeruginosa* CCTCC AB 2013115 were used as negative and positive controls, respectively. **(A)** Survival or clinical outcome of infected mice during the 2-d observation period. **(B)** Bacterial burdens in lung tissue and plasma after infection. **(C)** Representative H&E-stained lung sections showing inflammatory injury after infection. **(D)** Levels of TNF-α, IL-1β, and IL-6 in lung tissue or serum. Data are shown as mean ± SD. ns, not significant; *p < 0.05, **p < 0.01, ***p < 0.001.

Bacterial burdens in the lung, spleen, and plasma were next quantified to assess colonization and dissemination. Because most mice in the *P. aeruginosa* group died within 2 d, samples from dead mice were collected immediately on the day of death, and the remaining mouse was sampled at the experimental endpoint. *P. aeruginosa* reached 6.02 × 10^6^ CFU/g in lung tissue and was also recovered from the spleen at 2.22 × 10^6^ CFU/g (Supplementary Figure S1), indicating efficient lung colonization and dissemination to an immune organ. In contrast, all polar isolates showed markedly restricted colonization. At 1 and 2 d post-infection, viable bacterial loads of the polar isolates in the lung remained low, approximately 10^2^–10^3^ CFU/lung, and were not significantly different from those in the *E. coli* K-12 group (Figure 8B). No viable bacteria were detected in the spleens of mice infected with polar isolates, suggesting that these strains failed to expand efficiently *in vivo* or stably disseminate to the spleen. Notably, low levels of viable bacteria were detected in the plasma of mice infected with all polar isolates, with higher bacterial loads at day 1 than at day 2 post-infection (Figure 8B). This suggests transient bloodstream translocation rather than sustained systemic infection.

Histopathological analysis further showed that all polar isolates induced lung injury at 2 d post-infection. H&E-stained lung sections exhibited inflammatory cell infiltration, alveolar exudation, and thickening of the alveolar septa. Lung pathology scores in the polar-isolate groups were higher than those in the *E. coli* K-12 group but lower than those in the *P. aeruginosa* group (Figure 8C). Although ExlBA had been identified as a key factor for hemolysis and cytotoxicity, the corresponding Δ*exlB* mutants still induced mild lung inflammation, with pathology scores higher than those of the *E. coli* K-12 group (Supplementary Figure S2). This finding is consistent with the nematode assay, in which Δ*exlB* mutants retained partial lethality, and suggests that additional secreted factors or metabolic products may contribute to host injury.

Consistent with the histopathological findings, cytokine analysis showed that infection with polar isolates significantly increased the levels of TNF-α, IL-1β, and IL-6 in lung tissue, indicating a clear local acute inflammatory response (Figure 8D). In contrast, systemic cytokine responses were limited, with a significant increase in serum IL-1β detected only in the NS21-infected group (Figure 8D, Supplementary Figure S3). Together, these results indicated that although polar *Pseudomonas* isolates have limited colonization and systemic dissemination capacity in mice, they can transiently enter the bloodstream and induce local lung inflammation and tissue injury.

## 4 Discussion

One of the major findings of this study is the demonstration that psychrophilic *Pseudomonas* are capable of hemolysis, and this activity depends on the ExlBA system. ExlA has previously been recognized mainly as an important pore-forming toxin in T3SS-negative lineages of *P. aeruginosa* (Elsen et al., 2014). Here, the functional relevance of this system is extended to psychrophilic polar *Pseudomonas*, suggesting that ExlBA may not be restricted to clinically associated strains but may also act as a functional module involved in membrane damage and ecological competition in environmental pseudomonads. An important question raised by these findings concerns the ecological function of hemolytic activity in polar *Pseudomonas*. In clinical contexts, hemolysins are conventionally viewed as host-damaging virulence factors; in environmental bacteria, however, membrane-damaging and pore-forming activities are increasingly recognized as multifunctional traits that may serve nutrient acquisition, intermicrobial competition, and protection against grazing predators. Pore-forming toxins of the ShlA/ExlA family can lyse eukaryotic cells and thereby release nutrients, and hemolytic, siderophore-producing, and exoenzymatic phenotypes recovered from Antarctic soils have been interpreted as components of an ecological fitness repertoire that predates any clinical relevance (Acuña et al., 2026; Hertle et al., 1999). Consistent with this view, the polar isolates examined here produced siderophores and displayed membrane-damaging activity toward erythrocytes, cultured mammalian cells, nematodes, and mouse lungs, yet failed to colonize a mammalian host efficiently. Their hemolytic capacity may therefore have evolved primarily in the context of microbial competition and nutrient scavenging in cold, oligotrophic soils, and may confer only restricted host-interaction potential when warming-associated dispersal brings these bacteria into contact with warmer, host-associated environments. This ecological framing reconciles the presence of a functional pore-forming toxin in strains that are not host-adapted pathogens, and it underscores that “virulence-associated” phenotypes of polar microorganisms should be interpreted in their environmental context rather than by clinical analogy alone.

Unlike the pattern commonly observed in typical human pathogens, in which virulence expression is enhanced at host temperature (Roop et al., 2014), ExlBA-associated hemolysis in 3S6 and NS21 was attenuated after temperature elevation (Figures 4A,B), accompanied by significant decreases in the transcript levels of *exlA* and *exlB* (Figure 4C). Because that the transcriptional activator Vfr requires cAMP binding for functional activation (Almblad et al., 2015; Fuchs et al., 2010; Jin et al., 2019), the concurrent temperature-dependent upregulation of *cpdA* (2.43-fold in 3S6 and 6.36-fold in NS21 at 28 °C), the modest induction of the cAMP-synthesizing adenylate cyclase *cyaA* (1.27- and 1.61-fold, respectively), and the net 1.88- to 3.11-fold decrease in intracellular cAMP levels (Figures 4C,D) are most parsimoniously explained by enhanced cAMP turnover at elevated temperature. If increased cAMP synthesis were the predominant response, the cAMP pool would be expected to expand rather than contract; the observed depletion thus points toward enhanced degradation, consistent with the pronounced induction of *cpdA*. These observations support, but do not establish, a model in which enhanced CpdA-mediated cAMP hydrolysis at 28 °C reduces the availability of active cAMP-Vfr complexes, thereby attenuating *exlBA* transcription and hemolytic output. However, since CpdA enzymatic activity and cAMP degradation kinetics were not directly measured, and since transcriptional upregulation of *cpdA* does not necessarily reflect increased phosphodiesterase activity, direct functional validation, such as *cpdA* deletion or exogenous cAMP supplementation, remains required to conclusively establish causality.

For cold-adapted or psychrophilic microorganisms, 20 °C may fall within a permissive range for growth and metabolism, whereas 28 °C may impose thermal stress. Under this condition, reduced ExlBA-associated hemolysis may reflect a stress-associated reallocation of cellular resources. By downregulating energetically costly virulence proteins, bacteria may prioritize basal survival, membrane homeostasis, or other adaptive processes (Pruneau et al., 2018). Thus, warming does not necessarily enhance the virulence of polar microorganisms. Instead, virulence-associated traits may show trait-specific responses to temperature shifts. Risk assessment of polar microorganisms under climate change should therefore consider not only the presence of virulence genes, but also the expression and functional activity of specific virulence phenotypes across relevant temperature ranges.

In addition to hemolysis, 3S6 and NS21 retained several virulence-associated traits linked to environmental adaptation and host interaction. Although neither polar isolate exhibited detectable twitching motility or pyocyanin production, and both showed significantly weaker biofilm formation and siderophore production compared with the opportunistic pathogen *P. aeruginosa* CCTCC AB 2013115 (Figures 2A,B), these results indicate that 3S6 and NS21 do not lack virulence-associated functions, but their virulence profiles more closely resemble partially retained environmental opportunistic modules rather than the complete, efficient, and coordinated virulence systems characteristic of typical pathogens.

Beyond direct regulation of ExlBA expression, elevated temperature also induced broader physiological changes in strain 3S6. Transcriptomic analysis revealed widespread downregulation of genes involved in chemotaxis, motility, adherence, and nutritional or metabolic functions, accompanied by increased expression of biofilm-associated genes (Figure 5D). Phenotypic assays further confirmed reduced swarming and swimming motility together with enhanced biofilm formation at 28 °C (Figures 5E–H). These findings suggest that thermal stress may trigger a lifestyle transition from an actively motile state toward a persistence-associated biofilm state. Biofilm formation is energetically costly and often requires substantial cellular investment in extracellular matrix production and maintenance (Gheorghita et al., 2023; Liu et al., 2025). Although the underlying mechanisms remain unclear, the observed reduction in ExlBA-mediated hemolysis under elevated temperature is consistent with enhanced biofilm formation, which favors survival and persistence. This may represent an adaptive strategy for psychrophilic bacteria encountering warming conditions outside their optimal thermal niche.

Nevertheless, 3S6 and NS21 still exhibited measurable cell-damaging potential under host-relevant thermal conditions (Figures 6B,C). Thus, even though ExlBA expression is attenuated at elevated temperature, polar *Pseudomonas* still retains membrane-damaging activity after exposure to host temperature. *In vivo* models further supported this “limited but non-negligible” infection potential. In the nematode model, both 3S6 and NS21 significantly shortened the lifespan of *C. elegans*, although their lethality was weaker than that of the *P. aeruginosa* positive control. The lethality of NS21Δ*exlB* was significantly attenuated (Figure 7C), indicating that ExlBA makes an important contribution to NS21 virulence in the nematode model. In contrast, no significant difference was observed between 3S6Δ*exlB* and wild-type 3S6 (Figure 7B), suggesting that the contribution of ExlBA to *in vivo* virulence is strain-specific or context-dependent. Notably, the Δ*exlB* mutants still retained greater lethality than OP50, indicating that ExlBA is not the sole determinant of virulence. Other secreted factors, metabolic products, or immunostimulatory cell-structure-associated components may also contribute to host damage.

In the acute mouse model, the polar isolates behaved very differently from the lethal, disseminating *P. aeruginosa* control: no mortality occurred, lung burdens remained low (approximately 10^2^–10^3^ CFU/lung), and no bacteria were recovered from the spleen (Figure 8B). Nonetheless, low numbers of viable bacteria were transiently detected in plasma, and infected mice developed clear local lung injury-inflammatory cell infiltration, alveolar exudation, and alveolar septal thickening-together with elevated pulmonary TNF-α, IL-1β, and IL-6 and only limited serum cytokine responses (Figures 8C,D). These results corroborate the nematode data in showing that, although the polar isolates cannot efficiently colonize or disseminate in a mammalian host, they retain the capacity to cause local tissue injury and transient bloodstream entry upon host contact.

Taken together, this study proposes a cautious and data-consistent risk framework in which 3S6 and NS21 should be regarded as temperature-responsive environmental opportunists rather than established pathogens. Their potential risk does not arise from sustained systemic infectivity, but from three main features. First, they carry and express membrane-damaging factors such as ExlBA. Second, they retain measurable host-damaging activity under permissive or transient host-relevant thermal conditions. Third, even when the core hemolytic factor is impaired, additional virulence-associated factors may still contribute to host damage and inflammation.

More broadly, these findings indicate that warming-associated temperature shifts do not simply enhance or suppress microbial virulence; rather, temperature can reshape the balance between environmental persistence and host interaction through trait-specific modulation of virulence-associated functions. The pronounced divergence between 3S6 and NS21 under identical thermal conditions further shows that thermal adaptation strategies can vary substantially even among closely related polar *Pseudomonas* strains, underscoring the difficulty of generalizing virulence responses across polar microorganisms. This framework avoids equating climate change directly with the emergence of new pathogens and instead emphasizes that temperature shifts may alter microorganism-host or microorganism-ecosystem interactions in trait-specific ways.

This study has several limitations. First, evidence supporting the CpdA-cAMP/Vfr regulatory pathway is mainly based on transcriptional analysis and intracellular cAMP quantification. Further experiments, including cpdA deletion, exogenous cAMP supplementation, and functional validation of Vfr, are required to establish causal relationships. Second, the mouse infection experiment employed an acute infection model with a relatively short observation window, allowing assessment of early lung inflammation and transient bloodstream entry but limiting evaluation of long-term colonization, bacterial clearance dynamics, and infection susceptibility in immunocompromised hosts. Third, this study focused on two representative cell-associated hemolytic strains, which may not fully capture the diversity of virulence responses among polar microorganisms. Future studies incorporating long-term warming simulations, host-associated exposure scenarios, and broader comparative analyses across diverse polar bacterial taxa will help clarify how climate-driven environmental change influences microbial virulence evolution and ecological risk. Ultimately, our findings suggest that warming-associated temperature shifts may not simply increase the pathogenicity of polar microorganisms, but rather reshape the balance between persistence and host interaction through trait-specific modulation of virulence-associated functions.

## 5 Conclusion

In conclusion, this study demonstrates that Antarctic *Pseudomonas* isolates 3S6 and NS21 harbor functional ExlBA-dependent membrane-damaging activity, but elevated temperature attenuates rather than enhances this trait, challenging the assumption that warming uniformly increases microbial virulence and highlighting the importance of trait-specific thermal regulation in polar microorganism risk assessment. Elevated temperature attenuated *exlBA* transcription and hemolysis in association with altered cAMP metabolism and reduced intracellular cAMP levels, while promoting a shift from motility to biofilm formation in strain 3S6. Although these isolates induced host-cell damage, nematode lethality, and lung inflammation, they showed limited mammalian colonization and systemic dissemination. These results imply potential health risks of hemolytic polar *Pseudomonas*, and highlight the importance of evaluating microbial risk through phenotype-based assays under relevant environmental and host-contact conditions.

## Data Availability

The original contributions presented in the study are publicly available. This data can be found in the NCBI repository under accession numbers PRJNA1534163 and PRJNA1534160 and BioSample accession numbers: SAMN63433652 and SAMN63433622.
