# Supplementary material for: Hemolytic mechanism and virulence of Antarctic psychrophilic Pseudomonas
Source: Front Microbiol. 2026 Oct 6;17:1959947. doi: 10.3389/fmicb.2026.1959947 (PMC13680231; doi:10.3389/fmicb.2026.1959947)
Supplement: Supplementary file 1 [file Supplementary_file_1.DOCX]

Supplementary Figures and Tables

# 1 **Supplementary Figures and Tables**

## 1.1 **Supplementary Figures**

**Fig S1. Bacterial burdens of *P. aeruginosa* CCTCC AB 2013115 in lung, spleen, and plasma after infection.** Female BALB/c mice were intranasally infected with *P. aeruginosa* CCTCC AB 2013115 at a dose of 1×10⁷ CFU per mouse. Due to rapid mortality in this group, tissues and plasma were collected immediately from mice that died during the observation period, and the remaining mouse was sampled at the experimental endpoint. Bacterial burdens were determined by serial dilution and plate counting in lung tissue, spleen tissue, and plasma. Data are shown as mean±SD. ns, not significant; * *p*<0.05, ** *p*<0.01, *** *p*<0.001.

**Fig S2. Histopathological scores of lung tissues after acute infection.**

Lung histopathological changes were evaluated based on H&E-stained sections collected at 2 d post-infection. Lung injury was scored according to five pathological parameters: hemorrhage, exudation, and mucus accumulation; alveolar septal thickening and consolidation; recruitment and infiltration of inflammatory immune cells; emphysema; and pulmonary edema. Each parameter was graded on a 0-3 scale, where 0 indicated no pathological change, 1 indicated mild change, 2 indicated moderate change, and 3 indicated severe change. The total histopathological score was calculated by summing the scores of all five parameters.

**Fig S3. Serum TNF-α and IL-6 levels after acute infection.**

Serum levels of TNF-α and IL-6 were measured at 2 d post-infection to evaluate systemic inflammatory responses after infection with control strains, polar *Pseudomonas* isolates, and their corresponding Δ*exlB* mutants. Cytokine concentrations were determined by ELISA. Data are shown as mean±SD. ns, not significant; * *p*<0.05, ** *p*<0.01, *** *p*<0.001.

## 1.2 **Supplementary Tables**

**Table S1. Bacterial strains used in this study**

| **Strains** | **Description** | **Source** |
| --- | --- | --- |
| ***Escherichia coli*** |  |  |
| K-12 | negative control | CCTCC^a^ |
| OP50 | negative control | SunyBiotech |
| DH5α | F– φ80*lacZ*ΔM15 Δ(*lac*ZYA*-arg*F) U169 *rec*A1 *end*A1 *hsd*R17(r_k_–, m_k_+) *sup*E44λ-*thi-*1 *gyr*A96 *rel*A1 *pho*A | Gimmico |
| S17-1(λpir) | RP4-2(Km::Tn7, Tc::Mu-1), pro-82, λpir, *rec*A1, *end*A1, *thi*E1, *hsd*R17, *cre*C510 | Angyubio |
| ***Pseudomonas*** |  |  |
| *P. antarctica* 3S6 | Wild-type strain | This lab |
| 3S6Δ*exlB* | *exlB* knockout mutant of 3S6 | This study |
| 3S6Δ*exlB*+*exlB* | 3S6Δ*exlB* complemented with *exlB* | This study |
| *P. yamanorum* NS21 | Wild-type strain | This lab |
| NS21Δ*exlB* | *exlB* knockout mutant of NS21 | This study |
| NS21Δ*exlB*+*exlB* | NS21Δ*exlB* complemented with *exlB* | This study |

a, China Center for Type Culture Collection

**Table S2. Plasmids used in this study**

| **Plasmids** | **Description^b^** | **Source** |
| --- | --- | --- |
| pK18mobsacB (pK18) | Suicide vector for gene deletion in *Pseudomonas*, Km^r^ | Schafer et al. (1994) |
| pK18-3S6(Δ*exlB*) | Suicide vector for *exlB* deletion in 3S6 | This study |
| pK18-NS21(Δ*exlB*) | Suicide vector for *exlB* deletion in NS21 | This study |
| pBBR1MCS-2 (pBBR2) | Broad-host-range shuttle vector for gene cloning and expression in *Pseudomonas*, Km^r^ | Kovach et al. (1995) |
| pBBR2-3S6(*exlB*) | 3S6 *exlB* gene cloned into pBBR2 | This study |
| pBBR2-NS21(*exlB*) | NS21 *exlB* gene cloned into pBBR2 | This study |

b, Km: kanamycin.

**Table S3. Primers used in this study**

| **Primer** | **Sequence(5′→3′)^c^** | **Description** |
| --- | --- | --- |
| 27F | AGAGTTTGATCCTGGCTCAG | 16S rRNA gene |
| 1492R | GGTTACCTTGTTACGACTT | 16S rRNA gene |
| rpoD_F | AAAGCGCAACAGCAGTCTCG | *rpoD* gene |
| rpoD_R | TCACTCGTCGAGGAAGGAGCG | *rpoD* gene |
| RT_3S6_*rpoD*_F | TCACTCGTCGAGGAAGGAGCG | Real-time PCR |
| RT_3S6_*rpoD*_R | ATCGACATGAATACCGACCACAC | Real-time PCR |
| RT_NS21_*rpoD*_F | GCGTTGGCAGCGGTCG | Real-time PCR |
| RT_NS21_*rpoD*_R | TGCGCCCATCACTTCACG | Real-time PCR |
| RT_3S6_*exlA*_F | GGCGTCGTCCTGTTTCTGG | Real-time PCR |
| RT_3S6_*exlA*_R | ACCAGGGCAAGGTCGAC | Real-time PCR |
| RT_NS21_*exlA*_F | CGCCTGCAACTCCTGGC | Real-time PCR |
| RT_NS21_*exlA*_R | GTCGGGCTGAACGTCTCG | Real-time PCR |
| RT_3S6_*exlB*_F | GCTCAGGTTGAGCCCGAC | Real-time PCR |
| RT_3S6_*exlB*_R | GTGCGGTATGGCGCAACAC | Real-time PCR |
| RT_NS21_*exlB*_F | GTATTCACGCTTGACCCAGCC | Real-time PCR |
| RT_NS21_*exlB*_R | CACTGACGATTCGGCGG | Real-time PCR |
| RT_3S6_*Vfr*_F | ACCGCTCAGGGCGTAAAG | Real-time PCR |
| RT_3S6_*Vfr*_R | GAGCTGGGGCTATTTGAACAGG | Real-time PCR |
| RT_NS21_*Vfr*_F | CAAGGAACAGGAGCGCAGC | Real-time PCR |
| RT_NS21_*Vfr*_R | GTGTCTCTCAGGCGCTGG | Real-time PCR |
| RT_3S6_*CyaA*_F | GGTGAGCATTTCCAGCAGGTC | Real-time PCR |
| RT_3S6_*CyaA*_R | ACTTAGCCGAAGACACCCTGAC | Real-time PCR |
| RT_NS21_*CyaA*_F | CTGCTGCCGGGCTATGT | Real-time PCR |
| RT_NS21_*CyaA*_R | CAGGCCATGAATCGGCCG | Real-time PCR |
| RT_3S6_*CpdA*_F | CACATGCCCCCAGAGCAC | Real-time PCR |
| RT_3S6_*CpdA*_R | AAGCGCCGAGCCAACAC | Real-time PCR |
| RT_NS21_*CpdA*_F | CTGGATCGGTTTCCTCAGGTG | Real-time PCR |
| RT_NS21_*CpdA*_R | CTGCTCACTCACCTTGAAATCCT | Real-time PCR |
| pK18_MCS_F | CCGCCTCTCCCCGCGCGTTG | pK18 vector verification |
| pK18_MCS_R | GCGTGAAGCTAGCTTATCGCC | pK18 vector verification |
| pK18_sacB_F | CACATATACCTGCCGTTCACTATTATTTAGTGAAA | deletion mutant strains  verification |
| pK18_sacB_R | GTATGTTTCCTTATATGGCTTTTGGTTCGTTTCTT | deletion mutant strains  verification |
| pBBR2_MCS_F | TAGGCACCCCAGGCTTTACAC | pBBR2 vector verification |
| pBBR2_MCS_R | GGGTAACGCCAGGGTTTTCC | pBBR2 vector verification |
| 3S6_*exlB*_up_F | CGCGGATCCTTGAGGATCACGCTCGCCG | pK18-3S6Δ*exlB* construction |
| 3S6_*exlB*_up_R | GTGCCGAATCAGCTACAGATATGAGCAAACACCTGTTTAGC | pK18-3S6Δ*exlB* construction |
| 3S6_*exlB*_down_F | TCATATCTGTAGCTGATTCGGCACTCGACATCC | pK18-3S6Δ*exlB* construction |
| 3S6_*exlB*_down_R | CCCAAGCTTGGTAGGGATGGGCCCAGC | pK18-3S6Δ*exlB* construction |
| 3S6_*exlB*_F | TCATATCTGTAGGCTCAAGCGCG | 3S6Δ*exlB* verification |
| 3S6_*exlB*_R | GTGCCGAATCAGCCCTCAAC | 3S6Δ*exlB* verification |
| 3S6_*exlB*_ident_F | GCCAAAAATCTCCTGGGCGC | 3S6Δ*exlB* verification |
| 3S6_*exlB*_ident_R | AGCTGCGCAAGGCTACAG | 3S6Δ*exlB* verification |
| NS21_*exlB*_up_F | CCGGAATTCGATCACGCTCGCTGCCT | pK18-NS21Δ*exlB* construction |
| NS21_*exlB*_up_R | CTGCTCTGCCTGTTGCAGATATGACCCAACACCTGAT | pK18-NS21Δ*exlB* construction |
| NS21_*exlB*_down_F | TCATATCTGCAACAGGCAGAGCAGGGCC | pK18-NS21Δ*exlB* construction |
| NS21_*exlB*_down_R | CGCGGATCCCGATGAATCGATTCTGGTGCG | pK18-NS21Δ*exlB* construction |
| NS21_*exlB*_F | TCATATCTGCAAACTAAGGCGCG | NS21Δ*exlB* verification |
| NS21_*exlB*_R | CTGCTCTGCCTGTGCACG | NS21Δ*exlB* verification |
| NS21_*exlB*_ident_F | GCCAAAAATCTCCTGCGCAC | NS21Δ*exlB* verification |
| NS21_*exlB*_ident_R | CTGGATATCTTCGCCAAGGAAATGC | NS21Δ*exlB* verification |
| pBBR2_3S6_*exlB*_F | CCCAAGCTTATGCCGAATCAGCCCTCAAC | pBBR2-3S6(*exlB*) construction |
| pBBR2_3S6_*exlB*_R | CGCGGATCCTCATATCTGTAGGCTCAAGCGCGTCAG | pBBR2-3S6(*exlB*) construction |
| pBBR2_NS21_*exlB*_F | CCCAAGCTTATGCCGAATCAGCCCTCAAC | pBBR2-NS21(*exlB*) construction |
| pBBR2_NS21_*exlB*_R | CGCGGATCCTCATATCTGCAAACTAAGGCGCG | pBBR2-NS21(*exlB*) construction |

c, underlined sequences indicate restriction sites.
